# Is there a link between COVID-19 and obstructive sleep apnea?

**DOI:** 10.5935/1984-0063.20200078

**Published:** 2021

**Authors:** Cristina Salles, Juliana Rodrigues Lopes, Margarida Neves, Renata Silva Brito, Andrea Bacelar

**Affiliations:** 1 Escola Bahiana de Medicina e Saúde Pública, Pesquisa - Salvador - Bahia - Brazil; 2 Universidade Federal da Bahia - Salvador - Bahia - Brazil.; 3 Brazilian Sleep Association, President - Rio de Janeiro - Rio de Janeiro - Brazil.

**Keywords:** Coronavirus Infections, Obstructive Sleep Apnea, Sleep

## Abstract

Although obstructive sleep apnea syndrome (OSAS) is not considered a risk factor for COVID-19, studies have observed that these two conditions have comorbidities in common such as diabetes, cardiovascular diseases, asthma, obesity, hypertension, and chronic obstructive pulmonary disease. Thus, one may question the possible contribution of OSAS to the worsening of hypoxemia in patients with COVID-19 since OSAS and obesity (hypoventilation) are associated with hypoxemia, which can be a worsening factor in the hypoxemia of COVID-19 pneumonia. Moreover, one may question whether sleep deprivation would negatively interfere with the pulmonary condition caused by COVID-19. Another question would be whether sleep deprivation resulting from OSAS would be a favorable condition for the pulmonary inflammatory process in patients with COVID-19. Studies with a more significant number of participants are needed to assess the possible impact of OSAS on the outcomes of patients with SARS-CoV-2 infection, providing a more solid basis for making therapeutic decisions. An important advance in understanding the influence of OSAS on COVID-19 is represented by careful identification of comorbidities and potential pathophysiological mechanisms that may contribute to the favorable outcome of these patients.

## INTRODUÇÃO

A novel human coronavirus, called coronavirus of severe acute respiratory syndrome 2 (SARS-CoV-2), emerged in Wuhan, China, in late 2019 and is now causing a pandemic^1^. SARS-CoV-2 is considered being a respiratory virus capable of affecting both the upper and lower respiratory tracts. This syndrome has become a significant challenge to the healthcare community, governments, among other institutions^2^.

Once the pandemic started, there was a need for social isolation, which led to the suspension or marked reduction of regular sports activities, an increase in calorie intake, thereby promoting weight gain; besides, the literature reports that the most significant predictor of weight gain is this period has been self-reported anxiety/depression, which consequently contributes to the worsening of obstructive sleep apnea syndrome (OSAS)^3^. Studies have shown that about 70% of OSAS patients are obese and that the opposite is also true, i.e., 40% of obese patients have OSAS^4-6^. There is an association between abdominal circumference and apnea-hypopnea index so that a 13-15cm increase in abdominal circumference raises the risk for OSAS by about 4 times^7,8^. Hypoxemia and sleep fragmentation may be associated with increased inflammation in OSAS patients and may be identified by increased interleukin-6, C-reactive protein, tumor necrosis factor-alpha^9,10^. Besides this factor, the coexistence of chronic non-communicable diseases, such as obesity, in patients with COVID-19 may worsen and intensify the inflammatory process, and increase the risk of adverse outcomes and mortality^11^. The higher body mass index and excess adiposity carry risk factors for complications from COVID-19 infection. This condition may occur because of the higher prevalence of lung problems in obese populations compared to healthy weight counterparts^12^.

A marked increase of interleukin 1, IL-2, IL-4, IL-6, IL-8, and IL-10, the so-called “cytokine storm”^13^, characterized by the extensive and uncontrolled release of pro-inflammatory cytokines, can be observed in this disease. Clinically, the cytokine storm usually presents as systemic inflammation and multiple organ failure^14^. In addition to these changes, hypoalbuminemia, lymphopenia, neutropenia, and oxidative stress can be observed^15^.

**Figure 1:**
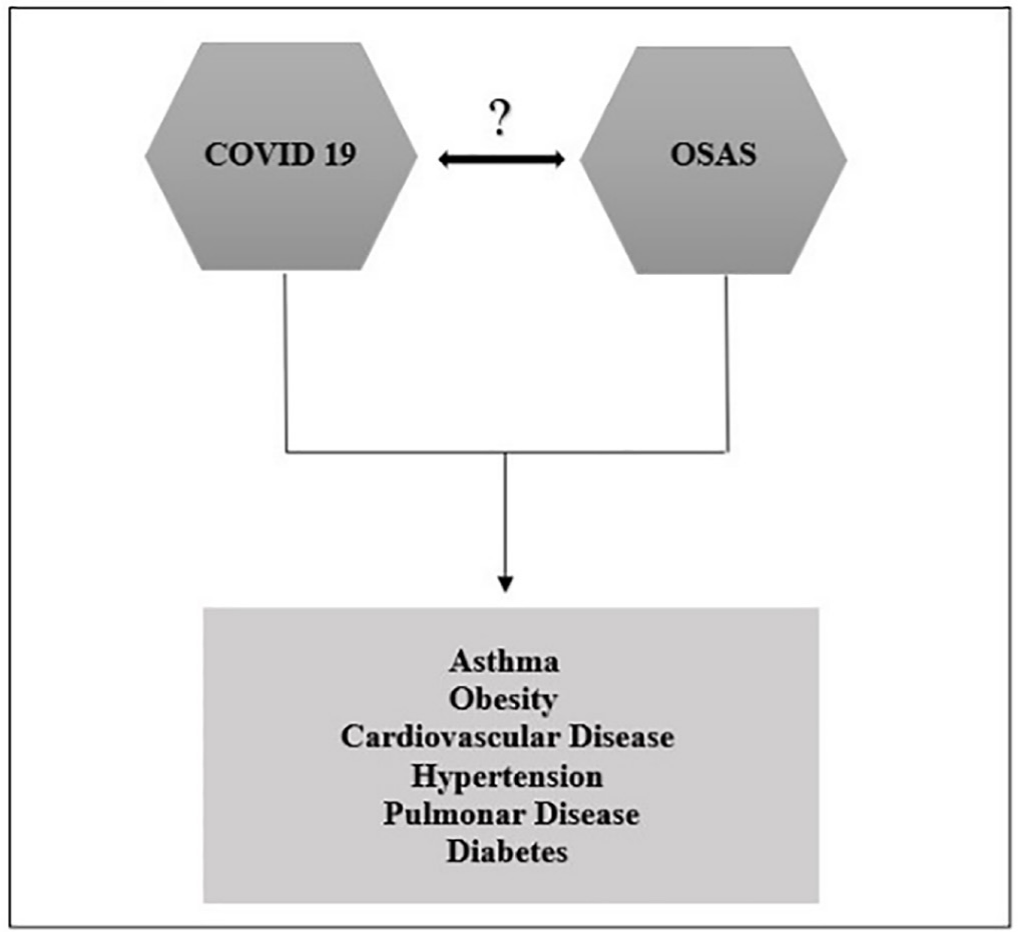
Common comorbidities between COVID-19 and OSAS

Although OSAS is not considered a risk factor for COVID-19, studies have shown the high frequency of this syndrome in intensive care unit patients due to the worsening of SARS-CoV-2 infection. McSharry and Malhotra (2020)^16^ draw attention to OSAS and COVID-19 because they have comorbidities in common such as diabetes, cardiovascular diseases, asthma, obesity, hypertension, and chronic obstructive pulmonary disease (Figure 1). These authors question about the possible contribution of OSAS to the worsening of hypoxemia in patients with COVID-19 since OSAS and obesity (hypoventilation) are associated with hypoxemia, which can be a worsening factor in the hypoxemia of COVID-19 pneumonia. On the other hand, they ask whether the benefits reported by early intubation in patients with COVID-19 could be related to OSAS improvement. No robust studies have been published yet to answer such questions. However, Bhatraju et al. (2020)^17^, when evaluating 24 patients with COVID-19 with a severe acute respiratory syndrome, in an intensive care unit (ICU) in Washington, found that 21% (5) presented OSAS, a value higher than that found for established comorbidities as a risk factor, e.g., asthma (14%) and chronic obstructive pulmonary disease (4%). Arentz et al. (2020)^18^ found a similar result in their study when they evaluated 21 ICU patients with COVID-19 and observed that among the comorbidities, 28.6% were diagnosed with OSAS, a higher frequency than that found with asthma (9.1%) and with the use of immunosuppressants (14.3%).

**Figure 2:**
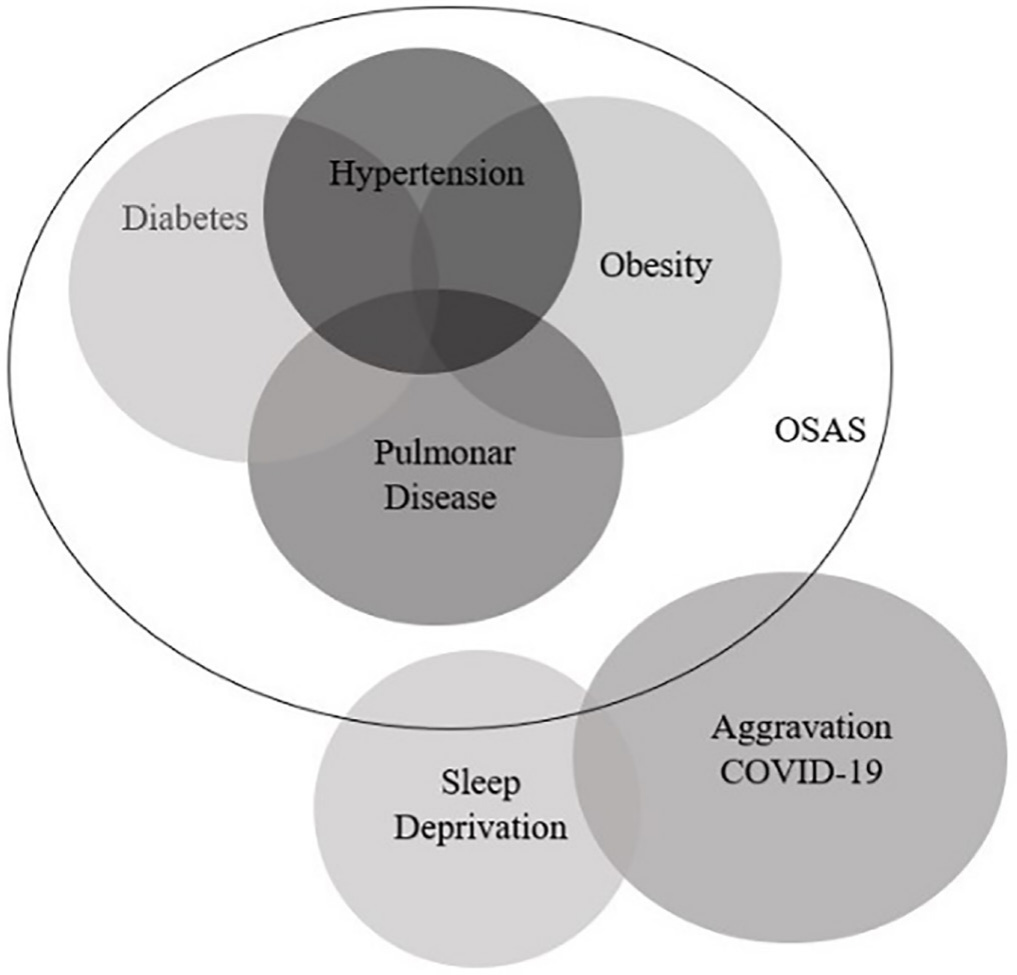
Association between comorbidities and aggravation COVID-19

During healthy sleep occurs the regulatory function of the immune response, with activation of T and B lymphocytes, release of pro- and anti-inflammatory cytokines^19^. However, patients with obstructive sleep apnea will present partial or complete obstruction of the upper airways, where airflow is decreased in hypopnea or completely interrupted in apnea, resulting in intermittent episodes of hypoxemia, hypercapnia, micro-awakenings and sleep fragmentation^20^. Therefore, patients with OSAS associated with sleep deprivation may present an increased production of inflammatory cytokines with marked neutrophilia. Nunes et al. (2018)^19^ conducted an experimental study comparing the pulmonary inflammatory response in mice exposed to the allergen (ovalbumin) that had a healthy sleep with those in sleep deprivation. They concluded that mice in sleep deprivation presented a greater inflammatory process in the airways than those with healthy sleep. Given this scenario, Salles (2020)^21^ questioned whether sleep deprivation would negatively interfere with the pulmonary condition resulting from COVID-19. Afterward, this question became more restricted, i.e., Salles and Barbosa (2020)^22^ questioned whether sleep deprivation resulting from OSAS would be a favorable condition for the pulmonary inflammatory process in patients with COVID-19 (Figure 2). Tufik et al. (2020)^23^ suggest the potential contribution of intermittent hypoxia observed in OSAS patients, which may interfere with the pulmonary ventilation, further compromising pulmonary parenchyma involvement, along with pulmonary vascular endothelial dysfunction resulting from the infectious response to SARS-CoV-2.

Nunes et al. (2018)^19^ observed that the anti-inflammatory response of the corticoid in mice in sleep deprivation and with an inflammatory process in the airways was more resistant than in those who had a healthy sleep, i.e., they were not able to modulate interleukin-17 and tumor necrosis factor-alpha production. On the other hand, the World Health Organization recommends the use of corticosteroids for the management of COVID-19. It has been proposed that corticosteroids would be beneficial in suppressing lung inflammation in severe acute respiratory syndrome because of their immunomodulatory properties^24^. Thus, we ask: could patients with OSAS and COVID-19 present greater resistance to corticoid treatment?

In line with the cited above, Grote et al. (2020)^25^ observed that sleep medicine specialists have been following in the opposite direction of COVID-19, i.e., sleep medicine services were reduced by almost 80% during the first 1-2 months of the pandemic in Europe. Second, polysomnography and PAP titrations have been wholly discontinued or practiced only to a limited extent in highly selected groups of patients. Third, the onset of sleep-disordered breathing (SDB) treatment with PAP therapy has also been reduced in most countries. Fourth, patient follow-up is now managed mainly by phone contacts. Fifth, the potential of mitigation strategies available through telemedicine has not been explored. Thus, the authors conclude that the sleep medicine community needs to collaborate in the development of strategies to assist patients with sleep-disordered breathing during major events such as the COVID-19 pandemic. Activities need to focus on the recognition of severe cases of SDR.

Studies with a more significant number of participants are needed to assess the possible impact of OSAS on the outcomes of patients with SARS-CoV-2 infection, providing a more solid basis for making therapeutic decisions. An important advance in understanding the influence of OSAS on COVID-19 is represented by careful identification of comorbidities and potential pathophysiological mechanisms that may contribute to the favorable outcome of these patients.

Thus, the present article demonstrates the need for further research considering the comparison of the severity of the clinical condition of individuals who were not diagnosed with OSAS but who were diagnosed with Covid-19 and those with OSAS and who were diagnosed with COVID-19. For better diagnostic elucidation, exclusion criteria could be presented in order to rule out confounding factors, i.e., comorbidities that could lead to a worse prognosis regarding COVID-19 infection (such as diabetes, systemic arterial hypertension, obesity, among other risk factors already established in the literature).
